# Protective role of *Ficus deltoidea* against BPA-induced impairments of the follicular development, estrous cycle, gonadotropin and sex steroid hormones level of prepubertal rats

**DOI:** 10.1186/s13048-018-0466-0

**Published:** 2018-11-26

**Authors:** Siti Sarah Mohamad Zaid, Shatrah Othman, Normadiah M. Kassim

**Affiliations:** 1 Department of Environmental Sciences, Faculty of Environmental Studies, Universiti Putra Malaysia, 43400 Serdang, Selangor Malaysia; 2 Department of Anatomy, University of Malaya, 50603 Kuala Lumpur, Malaysia; 3 Department of Molecular Medicine, Faculty of Medicine, University of Malaya, 50603 Kuala Lumpur, Malaysia

**Keywords:** *Ficus deltoidea*, BPA, Toxicity, Follicular, Estrous, Hormones, Ovary, Reproduction

## Abstract

*Ficus deltoidea* is one of the well-known medicinal plants in Malaysia that is traditionally used by the Malay community to treat various ailments and for maintenance of female reproductive health. The objective of this study is to evaluate the potential protective roles of *Ficus deltoidea* against BPA-induced toxicity of the pituitary-ovarian axis in pre-pubertal female rats. In this study, four groups of pre-pubertal female Sprague Dawley rats were administered with the followings by oral gavage for a period of six weeks: NC (negative control- treated with vehicle), PC (positive control-treated with BPA at 10 mg/kg/BW), F (treated with *Ficus deltoidea* at 100 mg/kg/BW, then exposed to BPA at 10 mg/kg/BW) and FC (*Ficus deltoidea* control - treated with *Ficus deltoidea* at 100 mg/kg/BW). Daily vaginal smear, ovarian follicular development as well as gonadotropin and sexual-steroid hormone levels were determined. The findings showed that *Ficus deltoidea* demonstrated preventive role against BPA-induced toxicity on the ovaries. This was evident by the increased percentage of rats with normal estrous cycle, qualitatively reduced number of atretic follicles (as observed in histopathological examination) and normalization of the gonadotropins hormone (FSH) and sexual steroid hormone (progesterone) levels. In conclusion, *Ficus deltoidea* has the capability to prevent the effects of BPA toxicity in the hypothalamus-pituitary-gonadal axis of prepubertal female reproductive system, possibly due to its variety of phytochemical properties. Therefore, these findings strongly support the traditional belief that this medicinal plant is beneficial as daily dietary supplement for the maintenance of female reproductive health.

## Introduction

In recent years, exposure to environmental toxicants has become a serious health concern. Anxiety over exposure to endocrine disrupting chemicals (EDCs) in humans and wildlife has escalated since they have detrimental effects on the development and functions of the reproductive system [1, 2]. One of the EDCs is the bisphenol A (BPA) that is widely used in the industries as plasticizer for the production of polycarbonate plastics and epoxy resins [3].

Process of reproduction in humans is tightly regulated by the hypothalamic-pituitary-gonadal axis (HPG) [4–6]. In the hypothalamus, secretion of gonadotropin releasing hormone (GnRH) is crucial for the coordination of reproductive maturation and activity. Meanwhile, pituitary gonadotropin hormones, namely FSH and LH, are directly influenced by GnRH. Hence, disruptions in the normal pulsatile secretion of GnRH by BPA in the brain consequently alter the level of gonadotropins secretion [7]. In the ovary, BPA exposure is reported to have negative effects on the granulosa cell steroidogenesis [8–10], reduces the pool of primordial follicles [11], increases antral follicles and reduces the percentage of corpora lutea [12]. BPA also increases the risk for development of polycystic ovaries syndrome (PCOS) [13] and decreases the levels of luteinizing hormone (LH) and follicle stimulating hormone (FSH) levels [14].

For these reasons, BPA may predispose these organs to earlier onset of diseases, reduced fertility and even promote cancer. Apart from the xenoestrogenic properties, the disruptive effects of BPA in the brain have been shown to be mediated by reactive oxygen species (ROS) generation. It is believed that the generation of ROS by BPA leads to the increase in the level of lipid peroxidation product (MDA) [15] while reducing the level of antioxidant glutathione (GSH). Thus, with these concerns in mind, we propose to use a natural product with high antioxidant activities, namely the *Ficus deltoidea,* as a potential therapeutic role to counter the deleterious effects of BPA on the reproductive system.

*Ficus deltoidea* is one of the most well-known and available *ficus* species in Malaysia; it is traditionally used by Malay community for health maintenance purposes particularly in the maintenance of female reproductive system [16]. This medicinal plant is locally known as Mas Cotek due to the tiny golden spots on the leaves. Extensive pharmacological studies have validated the traditional use of *Ficus deltoidea*, particularly for maintenance and fertility of the female reproductive system [17]. Apart from its beneficial effects on female reproductive system, this medicinal plant has also been reported to have antidiabetic [18], anti-inflammatory and anti-nociceptive [19], anti-melanogenic and anti-photoaging [20], anti-bacterial [21], wound healing [22], anti-cancer and cytotoxicity properties [23].

In the present study, systematic analysis on investigating the potential protective roles of *Ficus deltoidea* against the toxicity effects of BPA on the female reproductive system was conducted. Prepubertal female rats were concurrently treated with *Ficus deltoidea* and BPA by oral gavage over a six-week period. The protective effects of *Ficus deltoidea* on the follicular development, gonadotropin (17β-estradiol and progesterone) and sex steroid hormones (FSH and LH) as well as on the pattern of the estrous cycle were determined.

## Material and methods

### Animal and experimental design

All the experimental design and procedures were conducted according to the National Institutes of Health guide for the care and use of laboratory animals (NIH Publications No. 8023, revised in 1978) and approved by the Animal Care and Committee (ACUC) of University of Malaya. Female Sprague Dawley rats at 21-day of age were purchased from the Animal Husbandry Unit, Faculty of Medicine, University of Malaya. The rats were maintained under the standard laboratory conditions at 25 ± 2 °C, 50 ± 15% relative humidity and normal photoperiod of 12 h dark and 12 h light. They were acclimatized to the laboratory environment for a week before the commencement of the experiments. Tap water and commercial pellet diet (Gold Coin Feedmills Pte. Ltd., Malaysia) were supplied ad libitum*.* To minimize additional exposures to endocrine disruptors, all rats were placed in stainless steel cages with wood bedding and water was supplied in glass bottles. At 28-day of age, the rats were divided into four groups (*n* = 8) and treated as follows:

1. NC group (negative control) was treated with vehicle (0.2 ml of corn oil).
2. PC group (positive control) was treated with BPA suspended in vehicle at 10 mg/kg body weight.
3. F group (*Ficus deltoidea* + BPA) was treated with 100 mg/kg body weight of *Ficus deltoidea* 30 min before they were treated with BPA (10 mg/kg body weight).
4. FC group (*Ficus deltoidea* control) was treated with 100 mg/kg body weight of *Ficus deltoidea*.

The administration of the various treatment was performed once daily in the morning (between 09:00 and 10:00 AM) by oral gavage (to mimic the most likely route of human exposure) for six consecutive weeks. The daily body weight was recorded throughout the administration period. After the last treatment day, the rats were sacrificed during diestrous phase. Immediately, the weights of the ovaries were recorded, then ovaries were fixed in 10% buffered formalin for histopathological analysis.

### Aqueous extract of *Ficus deltoidea* (mas Cotek)

The female leaves of this medicinal plant were purchased from Herba Bagus Sdn. Bhd, Malaysia. The leaves were then deposited at the Herbarium of the Institute of Bioproduct Development (IBD), Universiti Teknologi Malaysia (UTM), Johor, after they were authenticated by a licensed botanist (voucher specimen: MFD 6). The International Plant Names Index is *Ficus deltoidea* Jack (ID no.: 852727). For aqueous extraction preparation, 10 kg of leaves were boiled with 80 L of distilled water for two hours. Then, the extract was filtered and freeze-dried at 180 °C (inlet temperature) and 108 °C (outlet temperature). Finally, the aqueous extract of *Ficus deltoidea* was kept in an air-tight container and stored in a refrigerator at 4 °C until use.

**Fig. 1:**
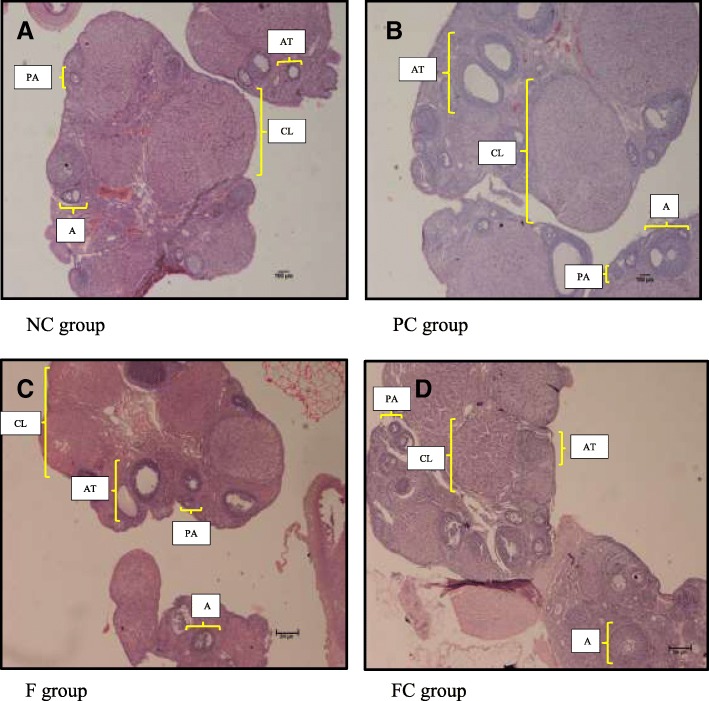
Representative histological sections from ovary rat of all experimental groups (H&E, 40×). **a** In NC group, normal histological appearance was observed. **b** More apparent atretic cystic-like follicles were observed in BPA-exposed rats (PC group). **c** Less atretic follicles were observed in BPA-exposed rats concurrently treated with *Ficus deltoidea* (F group). **d** The histological appearance in rats treated with *Ficus deltoidea* (FC group) was comparable to the control rats (FC group). **PA**: Preantral; **A**:Antral; **At**:Atretic; **CL**:Corpus Luteum. NC- Negative Control group (vehicle corn oil). PC- Positive Control group (BPA 10 mg/kg). F-*Ficus deltoidea* group (*Ficus deltoidea* 100 mg/kg + BPA 10 mg/kg). FC- *Ficus deltoidea* Control group (*Ficus deltoidea* 100 mg/kg)

The selection dose of 100 mg/kg body weight of aqueous extract of *Ficus deltoidea* was based on a previous subchronic toxicity study where no signs of toxicity effects were observed in hematological and biochemical parameters in rats [24]. *Ficus deltoidea* female leaves were selected due to its higher antioxidants levels compared to the male leaves (Fig. 1), in accordance with the non-enzymatic and enzymatic antioxidant assays [25]. The extract of the leaves has also been found to be more potent in cytotoxicity activity than the fruit extract [26]. The dose of BPA at 10 mg/g body weight was based on previous studies that induced disruption on morphological and biochemical parameters in the reproductive system [27–30].

### Estrous cycle assessment

In the present study, the first day of daily vaginal smear was commenced once vaginal opening was detected in the rats (between 09:00 and 10:00 AM). For the collection of vaginal secretions, micropipette tips filled with approximately 0.2 ml of normal saline (NaCl 0.9%) was inserted into vagina to a depth of 2 to 5 mm. Normal saline was used as an isotonic solution with osmolarity closed to the blood that provide longer period for the cells from lysis. Then, the normal saline was flushed repeatedly into the micropipette tips for three times before the vaginal secretion sample was collected. A drop of suspension was smeared onto a labeled glass slide and immediately viewed under a light microscope, using objective lenses ×10 and ×40 (for determination of proportion the three cell types and characterization of dominant cells, respectively). Cytological appearance of estrous phase was determined as follows:

1. The proestrous phase (twelve to fourteen hours) consists of predominance of nucleated epithelial cells.
2. An estrous phase (twenty-five to twenty-seven hours) primarily consists of anucleated cornified cells.
3. An metestrous phase (six to eight hours) indicated by equal proportions of leucocytes, anucleated cornified and nucleated epithelial cells.
4. The diestrous phase (fifty-five to fifty-seven hours) primarily consists of predominance of leucocytes.

The estrous cycle patterns are described as follows:

1. Normal cycle: A 4 to 5-day of estrous cycle in which the estrous phase was observed at least twice during the sampling period.
2. Persistent diestrous: or prolonged diestrous that lasts 4 or more days of diestrous phase during most of the cycle.

### Histopathological evaluation

The formalin-fixed ovary was processed by dehydration in a graded series of ethanol, cleared by xylene and infiltrated in paraffin using an automatic tissue processor (Citadel 2000, Thermo-Scientific, USA). Subsequently, these processed tissue was embedded in paraffin. The ovary paraffin block was serially sectioned at 5 μm thickness, mounted onto labeled glass slides, deparaffinized in xylene, stained with hematoxylin and eosin (Sigma Aldrich, USA), and finally dehydrated in a graded series of ethanol, cleared in xylene and mounted with Canada Balsam (Sigma-Aldrich, USA).

### Classification and quantification of ovarian follicles

Classification and quantification of ovarian follicles were performed on 62 total field areas and measured with grid lines using NIS-elements software as described by Zhuang et al. [31]. One section was chosen out of every 20 sections with five sections collected from each ovary. Each of selected ovarian sections were traced around the tissue boundaries with the computerized software and a sampling grid is superimposed over the section. The standardized size of counting fields were manually traced on the gridlines

A) A primary follicle comprises an oocyte surrounded by a single layer of cuboidal granulosa cells while a secondary follicle consists of an oocyte surrounded by more than one layer of cuboidal granulosa cells and no visible antrum.
B) An antral follicle has a clearly defined antral space and cumulus granulosa cell layer.
C) The corpus luteum is comprised of lutein cells and formed only after ovulation.
D) An atretic follicle is a degenerated follicle with inspissated follicular fluid, degenerated oocyte, disorganized and thickened granulosa cell layers or filled with fibrinous material in the antrum.

### Serum FSH, LH, 17β-estradiol and progesterone assay

Measurement of these hormone levels were performed using ELISA based kits (Cusabio, USA) following similar principal and protocols. Each hormone was analyzed using different kits according to the specific Antibody and Horseradish Peroxidase.

In brief, 50 μl duplicates of standards, samples and blank were added to the representative wells coated with E_2._ Subsequently, 50 μl of specific HRP conjugate were added to each well (except to blank wells). Then, 50 μl of specific antibody for each hormone was added to each well. The solutions in the wells were mixed and incubated for two hours in a 37 °C incubator (Echo Thermo, USA). After the incubation period, these mixed solutions were carefully aspirated from each well and the wells were rinsed three times using Wash Buffer solutions. Subsequently, 50 μl of the Substrate A and Substrate B supplied in the kit were added to each well and incubated again for another 15 min at 37 °C.

Finally, the reactions were terminated using 50 μl of Stop Solution. A microplate reader (BioTek, USA) was used to measure the optical density (OD) at wavelength of 450 nm. A standard curve was constructed by plotting a graph of the absorbance of each reference standard against its corresponding level and used to determine each hormone level.

### Statistical analysis

All statistical analysis was performed used Statistical Package for Social Sciences (SPSS Inc. Chicago, Illinois, USA version 18.0 for windows). Results were analyzed using two-way analysis of variance (ANOVA). Values are reported as mean ± S.E.M. *P* < 0.05 was considered significant.

## Results

### Estrous cycle

As shown in Table 1, all rats in the NC and FC groups had normal estrous cycles (100%). However, for BPA-exposed rats (PC group) only 12.5% had normal estrous cycles, while majority of rats were in persistent diestrous phase; these estrous cycle patterns were significantly different compared to the NC and PC groups. In BPA-exposed rats concurrently treated with *Ficus deltoidea* showed better percentage of rats with normal estrous cycles (62.5%) and a reduction of rats in persistent diestrous phase compared to BPA-exposed rats without treatment (PC group).

**Table 1:** Effect of BPA and *Ficus deltoidea* on the estrous cycle (*n* = 8)

Group	Rat with normal estrous cycle, n (100%)
NC	8/8 (100%)
PC	1/8^a^ (12.5%)
F	5/8^b^ (62.5%)
FC	8/8 (100%)

Data are expressed as Mean ± SEM

NC vs. PC = ^a^

PC vs. F = ^b^

*NC* Negative Control group (vehicle corn oil)

*PC* Positive Control group (BPA 10 mg/kg)

*F Ficus deltoidea* group (*Ficus deltoidea* 100 mg/kg + BPA 10 mg/kg)

*FC Ficus deltoidea* Control group (*Ficus deltoidea* 100 mg/kg)

### Body weight and organ weight

A slightly increment of body weight gain was observed in rats exposed to BPA (PC group) compared to the control rats (NC group) as shown in Table 2. Meanwhile, the body weight gain of rats treated with *Ficus deltoidea* alone (FC group) were comparable to those of the control rats (NC group).

**Table 2:** Effect of BPA and Ficus deltoidea on body weight and weights of ovary of rats (n = 8)

Group	Body weight at sacrifice (g)	Body weight gain (g)	Changes in body weight gain (%)	Ovarian wet weight (mg)	Relative weight of ovary (wet weight/body weight)
NC	260.22 ± 6.61	78.88 ± 4.61	48.15 ± 2.60	36.88 ± 1.88	0.24 ± 0.01
PC	287.31 ± 5.90	99.25 ± 3.90	56.52 ± 3.49	49.38 ± 1.13	0.29 ± 0.01^a^
F	280.29 ± 4.62	92.50 ± 2.62	56.12 ± 1.94	35.00 ± 1.34	0.21 ± 0.01^b^
FC	272.89 ± 4.64	89.50 ± 2.64	52.39 ± 3.47	33.13 ± 2.49	0.20 ± 0.02

Data are expressed as Mean ± SEM

NC vs. PC = ^a^

PC vs. F = ^b^

*NC* Negative Control group (vehicle corn oil)

*PC* Positive Control group (BPA 10 mg/kg)

*F Ficus deltoidea* group (*Ficus deltoidea* 100 mg/kg + BPA 10 mg/kg)

*FC Ficus deltoidea* Control group (*Ficus deltoidea* 100 mg/kg)

As depicted in Table 2, rats exposed to BPA showed significantly higher relative ovary weight compared to the control rats (NC group). This increment was significantly prevented by concurrent treatment with *Ficus deltoidea* (F group). The relative ovary weight in rats treated with *Ficus deltoidea* alone (FC group) was slightly lower but not significantly different when compared to the control rat (NC group).

### Hormonal profiles

As shown in Table 3, serum FSH and LH levels were significantly reduced in BPA-exposed rats (PC groups) compared to the control rats (NC group). Meanwhile, in BPA-exposed rats concurrently treated with *Ficus deltoidea* had significantly higher FSH levels. On the other hand, reduction of LH level in BPA-exposed rats was not significantly prevented by *Ficus deltoidea* treatment. Both these hormonal levels were comparable in rats from FC and NC groups.

**Table 3:** Level of reproductive hormones in all experimental groups (n = 8)

Group	Follicle Stimulating Hormone (FSH) (mIU/ml)	Luteinizing Hormone (LH) (mIU/ml)	17β-Estradiol (pg/ml)	Progesterone (ng/ml)
NC	78.50 ± 4.96	10.25 ± 2.71	19.04 ± 3.99	64.65 ± 3.28
PC	31.25 ± 5.96^a^	1.99 ± 0.32^a^	24.46 ± 2.22	34.95 ± 4.56 ^a^
F	52.87 ± 2.34^b^	1.91 ± 0.12	22.42 ± 2.07	42.78 ± 3.36 ^b^
FC	72.00 ± 7.25	9.30 ± 1.19	21.42 ± 1.62	52.16 ± 4.57

Data are expressed as Mean ± SEM

NC vs. PC = ^a^

PC vs. F = ^b^

*NC* Negative Control group (vehicle corn oil)

*PC* Positive Control group (BPA 10 mg/kg)

*F Ficus deltoidea* group (*Ficus deltoidea* 100 mg/kg + BPA 10 mg/kg)

*FC Ficus deltoidea* Control group (*Ficus deltoidea* 100 mg/kg)

In BPA-exposed rats (PC group), the level of E_2_ was slightly higher than the control rats (NC group). Comparable increment of E_2_ level was observed in BPA-exposed rats concurrently treated with *Ficus deltoidea* (F group) but at a lower magnitude compared to the rats exposed to BPA alone. E_2_ level in rats treated with *Ficus deltoidea* alone (FC group) was comparable to the normal control rats (NC rats).

Progesterone level in BPA-exposed rats was significantly lower compared to the control rats (NC group). However, progesterone level in BPA-exposed rats concurrently treated with *Ficus deltoidea* was significantly reduced. Meanwhile, the progesterone level in *Ficus deltoidea* treated rats was comparable to the normal control rats (NC group).

### Ovarian follicular counting

As depicted in Fig. 1, all stages of follicular development that consisted of preantral, antral, corpus luteum and atretic follicles were present in the ovaries of rats from all experimental groups. Control (NC group) and *Ficus deltoidea* treated alone (FC group) rats showed normal histological characteristics of ovaries. As for the PC and F groups, different histological findings were noted. Histological abnormalities of the ovaries include large antral-like follicle and atretic cystic-like follicles that did not reach ovulation stage. Accordingly, less number of corpora lutea were found in both groups as a result of reduced follicles reach ovulation stage. The degree of abnormalities was more apparent in the ovaries of BPA-exposed rats alone (PC group) compared to the BPA-exposed rats concurrently treated with *Ficus deltoidea* (F group).

**Fig. 2:**
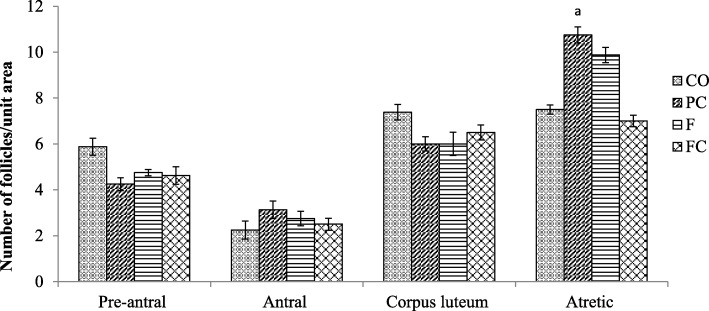
Number of follicles in all experimental groups. In BPA-exposed rats (PC group), the number of atretic follicles was significantly higher compared to the control rats (NC group). *Ficus deltoidea* was concurrently treated in the BPA-exposed rats (F group) and slight reduction was observed the number of atretic follicles compared to the BPA-exposed to *Ficus deltoidea* alone. Data are expressed as Mean ± SEM. 1) ^a^P < 0.05 vs. NC. 2) ^b^P < 0.05 and ^bb^P < 0.01 vs. PC. 3) ^d^P < 0.05 and ^dd^P < 0.01 vs. FC. NC- Negative Control group (vehicle corn oil). PC- Positive Control group (BPA 10 mg/kg). F-*Ficus deltoidea* group (*Ficus deltoidea* 100 mg/kg + BPA 10 mg/kg). FC- *Ficus deltoidea* Control group (*Ficus deltoidea* 100 mg/kg)

Further quantitative analysis of the follicular number in all ovaries from all experimental groups were conducted to determine the degree of these ovarian histological derangement (Fig. 2). In general, consistent results of the ovarian histological changes and follicular counts were observed. Control (NC group) and *Ficus deltoidea* treated alone (FC) rats have shown comparable morphology of all the ovarian follicles (preantal, antral, corpus luteum and atretic follicles) with the histological findings of normal ovaries.

Disruptive histological changes in rats from PC group were consistent with the follicular count. In BPA-exposed rats (PC group), the number of atretic follicles was significantly higher compared to the control rats (NC group). Even not statistically significant, there were slightly lesser atretic follicle count in BPA-exposed rats concurrently treated with *Ficus deltoidea* (F group). The higher number of atretic follicles in BPA-exposed rats could be related to the histological findings where more apparent atretic cystic-like follicles were observed compared to the rats concurrently treated with *Ficus deltoidea* and BPA (F group).

## Discussion

BPA is one of the EDCs that is reported to induce weight gain in rodent models [31, 32]. In the present study, the mean body weight of BPA-exposed rats were slight increased, but was not significantly different compared to the control rats. This suggests that BPA may not be directly correlated with weight gain and this is in agreement with previous reports [33–35]. A study by Kwon et al [33] found that oral administration of BPA at high doses had no effect on the body weight of rats. Similar findings from another study also reported no difference in the body weight of rat offspring that were perinatally-exposed to low doses of BPA [36]. In fact, the discrepancy in the findings of body weight changes remain enigmatic and could be due to many factors such as differences in the sensitivity of the strain used, dose, route of exposure, window of exposure (age) and duration of exposure [37–39].

Many studies have reported on the disruptive effects of BPA on the estrous cycle of rats [9, 33, 40, 41]. Our findings were in agreement with these reports that the duration of estrous cycle was prolonged (more than 4 or 5 days) in BPA-exposed rats compared to the control rats. This was likely due to the long diestrous phase as shown in our results. The disruption of estrous cycle in BPA-exposed rats could be due to the alteration in the normal functions of the hypothalamic-pituitary axis, which interfered with the normal production of gonadotropin releasing hormone (GnRH) and thereby decreasing the secretion of FSH and LH levels. The interference by BPA on the production of gonadotropin hormones (FSH and LH) could affect the gonadal function, which consequently disrupts the production of sexual steroid hormones, namely E_2_ and progesterone from the ovary.

Concurrent treatment with *Ficus deltoidea* of BPA-exposed rats had resulted in significant increase in the percentage of rats with normal estrous cycle up to 62.5%. Theoretically, the increase in the percentage of normal estrous cycle in BPA-exposed rat should essentially be related to the hypothalamic-pituitary axis, where normalization of gonadotropins hormone levels ought to be apparent. Improvement in the percentage of normal estrous cycle could be associated with reduced harmful effects of BPA on the ovarian follicular development in BPA-exposed rats. However, in this case, the increase in the percentage of normal estrous cycle in BPA-exposed rats is only accompanied by an increase in the level of FSH but not LH, and the reasons for these remain enigmatic. It may be possible that *Ficus deltoidea* may not be affecting the pituitary, but acting primarily on the ovaries instead. They could be displaying their effects through estrogenic mechanisms via ERs at the ovaries, hence protecting the process of follicular development and ovulation. As we have shown, even with reduced LH level, ovulation still occurs as evidenced by the restoration of progesterone level secreted by corpus luteum in *Ficus deltoidea*-treated rats. Therefore, further investigation is warranted to define the mechanism that may be responsible to restore the normal estrous cycle in BPA-exposed rats treated with *Ficus deltoidea* without significantly affecting the hypothalamic-pituitary functions.

The ovary is the main target organs for xenoestrogenic compounds as proven in many scientific studies [13, 42, 43, 44]. In the present study, the weight of ovary of BPA-exposed rats was increased by 20% compared to the control rats. Based to this result, it appears that BPA has some influence on the weight of ovary, without having significant effects on the body weight. This finding supports a previous report by Ashby et al. [45]. In fact, any changes of 10% or greater in the organ weight is a strong indicator of toxic effects, particularly when it is associated with morphological changes. In this study, the significant changes in the organ weight and size of ovary of BPA-exposed rats were in fact coupled with the overall disruptive effects by BPA.

In contrast to the normal histology of the normal ovary of the control rats, the presence of large-antral and atretic-cystic like follicles in BPA-exposed rats were evident. These abnormal follicles reflected the pathological alteration in the secretion of gonadotropins hormone (decreasing levels of FSH) and possibly account for the increase in the ovarian weight.

Reduction in the LH level observed in the BPA-exposed rats may be the reason for the formation of cystic follicles (anovulatory follicles) and consequently reduction in the formation of corpora lutea in the ovary (formed after ovulation). When the formation of corpora lutea is reduced, the secretion of progesterone is low. Meanwhile, the increase number of cystic antral- like follicles with reduced corpora lutea in BPA-exposed rats in this study agrees with previous findings [9, 46]. Cystic follicles are large antral-like follicles surrounded by thin layer of granulosa cells with non-detectable theca cell layers that do not support ovulation process in the ovary [47, 48]. Interference with the normal development of follicles in the ovary was evidenced by reduction in the number of preantral follicles and increase in the number of atretic cystic-like follicles in BPA-exposed rats. Interestingly, changes in the follicular populations and histology in the ovary of BPA-exposed rats were similar to those seen in aging rats [49].

Changes in the relative ovarian weight of BPA-exposed rats were prevented by *Ficus deltoidea* treatments. The increase in ovarian weight, respectively, were normalized to the control levels. These suggest that *Ficus deltoidea* has the protective properties that prevent weight changes of the reproductive organ. Subsequently, further investigation showed that normalization of this organ weight by *Ficus deltoidea* was also correlated with the improvement of their morphology where the follicles were seen relatively healthier. These suggest the protective effects of *Ficus deltoidea* against the disruptive effects of BPA on the cellular components/level of the reproductive organ. The histological findings of ovaries rats treated with *Ficus deltoidea* alone or to the control rats, indicates that *Ficus deltoidea* has no pathological effects on this cellular organ. Although the morphological improvements in ovary were seen to be significant in BPA-exposed rats treated with *Ficus deltoidea*, the protection exerted by this medicinal plant seemed to be only partial compared to the control rats. This could be due to the incomplete elimination of ROS, thus enabling continual disruption on the cellular components and follicles. Moreover, even after elimination of ROS and BPA, the proteins/enzymes involved in the intracellular protective machinery may require a longer period of time to recover from the oxidative damage that has occurred [50]. Further analysis on this is therefore warranted.

## Conclusions

In the present study, we demonstrated significant protective effects of *Ficus deltoidea* against the toxicity caused by BPA in the reproductive system. This medicinal plant has the capabilities to prevent toxicity effects of BPA as shown by the increase in the percentage of rats with normal estrous cycle, increase in the level of gonadotropins hormone (FSH) and sexual steroid hormone (progesterone) as well as reduction in the formation of the atretic follicles (qualitatively observed under the histopathological examination).

The current findings strongly support the traditional belief that herbal plants are important as daily supplements in the diet for the promotion of long term reproductive health and fertility. In conclusion, *Ficus deltoidea* are able to prevent the toxicity effects of BPA on the prepubertal female reproductive system, possibly due to their variety of phytochemical properties. Hence, they may be particularly beneficial as health supplements to help in reducing the risk of permanent female reproductive infertility caused by prolonged BPA exposure, especially during their prepubertal period of life.

## Abbreviations

BPA: Bisphenol A
EDCs: Endocrine disrupting chemicals
F: Ficus + BPA group
FC: Ficus group
FSH: Follicle stimulating hormone
GnRH: Gonadotropin releasing hormone
GSH: Gluthione
HPG: Hypothalamic-pituitary-gonadal axis
LH: Luteinizing hormone
MDA: Malondialdehyde
NC: Control group
OD: Optical density
PC: BPA-exposed group
PCOS: Polycyctic ovaries syndrome
ROS: Reactive oxygen species
